# The Sit-to-Stand Transition as a Biomarker for Impairment: Comparison of Instrumented 30-Second Chair Stand Test and Daily Life Transitions in Multiple Sclerosis

**DOI:** 10.1109/TNSRE.2022.3169962

**Published:** 2022-05-16

**Authors:** Lindsey J. Tulipani, Brett Meyer, Samantha Fox, Andrew J. Solomon, Ryan S. McGinnis

**Affiliations:** Department of Electrical and Biomedical Engineering, University of Vermont, Burlington, VT 05405 USA; Department of Electrical and Biomedical Engineering, University of Vermont, Burlington, VT 05405 USA; Department of Electrical and Biomedical Engineering, University of Vermont, Burlington, VT 05405 USA; Department of Neurological Sciences, University of Vermont, Burlington, VT 05405 USA; Department of Electrical and Biomedical Engineering, University of Vermont, Burlington, VT 05405 USA

**Keywords:** Wearable, accelerometer, sit-to-stand, multiple sclerosis, falls

## Abstract

Falls and mobility deficits are common in people with multiple sclerosis (PwMS) across all levels of clinical disability. However, functional mobility observed in supervised settings may not reflect daily life which may impact assessments of fall risk and impairment in the clinic. To investigate this further, we compared the utility of sensor-based performance metrics from sit-stand transitions during daily life and a structured task to inform fall risk and impairment in PwMS. Thirty-seven PwMS instrumented with wearable sensors (thigh and chest) completed supervised 30-second chair stand tests (30CST) and underwent two days of instrumented daily life monitoring. Performance metrics were computed for sit-stand transitions during daily life and 30CSTs. EDSS sub scores and fall history were used to dichotomize participants into groups: pyramidal/no pyramidal impairment, sensory/no sensory impairment and high/low fall risk. The ability of performance metrics to discriminate between groups was assessed using the area under the curve (AUC). The feature that best discriminated between high and low fall risk was a chest acceleration measurement from the supervised instrumented 30CST (AUC = 0.89). Only chest features indicated sensory impairment, however the task was different between supervised and daily life. The metric that best discriminated pyramidal impairment was a chest-derived feature (AUC = 0.89) from supervised 30CSTs. The highest AUC from daily life was observed in faller classification with the average sit-stand time (0.81). While characterizing sit-stand performance during daily life may yield insights into fall risk and may be performed without a clinic visit, there remains value to conducting supervised functional assessments to provide the best classification performance between the investigated impairments in this sample.

## I. Introduction

Multiple sclerosis is an immune-mediated disease that causes inflammation, demyelination and neurodegeneration of the central nervous system leading to compromised nerve signaling between the brain and body. The disruption in signal transmission of the sensorimotor systems lead to a high incidence of balance deficits, mobility problems, and elevated fall risk in people with multiple sclerosis (PwMS) [1], [2] across all levels of disability [3]. As such approximately 50% of PwMS will fall in a given three-month period [4] and it has been shown that pyramidal and sensory impairment are greater in people who fall [5]. Balance is comprised of an integrated, multifactorial system inclusive of both pyramidal and sensory components, each contributing to functional mobility differently. Proprioceptive deficits of lower extremities occur in two-thirds of PwMS and are associated with poor awareness of where the limbs are in space [6]–[8]. Whereas pyramidal impairments are associated with spasticity and lower extremity strength deficits reflected in decrements of functional and walking capacity [9], [10].

While there is ample evidence that interventions across a spectrum of disability levels lead to improvements in balance, physical functioning and decreased fall risk [11]–[13], establishing specific contribution to impairment remains a challenge in the clinic as clinicians are currently limited by rudimentary tools and time to perform complex assessments during routine exams. Thus, clinicians continue to rely on patient reported outcomes and standardized functional assessments to guide patient care [14]–[17]; though a growing number of studies are demonstrating the utility of wearables for longitudinal monitoring, personalized intervention and preventative care [18]. Additionally, it has been shown that instrumenting standardized functional assessments with wearable sensors improves the discriminative ability to detect group differences and could provide useful objective measures of performance during clinic visits [19]–[22]. However, studies comparing laboratory to daily life performance agree that measurements from supervised settings may not directly translate to mobility impairment during daily life [23]–[27]. Many recent studies have focused on performance differences in the laboratory and free living contexts in people with neurological impairments during gait [24]–[26], [28], turns [23] and sit-stand transitions [29], [30]. While performance metrics of sit-stand transitions identified during daily life have been shown to discriminate between groups (neurological vs healthy controls) [29], [30], no previous study has directly compared supervised and unsupervised performance of sit-to-stand and stand-to-sit transitions to discriminate fall risk in PwMS nor compared daily life performance to a structured task (i.e., 30CST) to investigate specific contributions to impairment and fall risk. Clinicians may benefit from improving methods to detect and quantify underlying mechanisms that contribute to balance impairment and information from varied contexts could be useful for deploying personalized interventions to target specific impairments.

Thus, we posit that there may be benefits to both supervised and unsupervised assessments of functional mobility incorporating wearables. To our knowledge no previous study has directly compared supervised and unsupervised performance during a structured task to a similar task performed during daily life to investigate specific contributions to impairment and fall risk. In this study we identified sit-stand and stand-sit transitions performed during daily life during two days of unsupervised monitoring and compared performance metrics derived from wearable sensors to those from a supervised and unsupervised 30-second chair stand test (30CST). Specifically, we sought to understand how performance in each context may inform fall risk, sensory and pyramidal impairment.

## II. Methods

### A. Participants and Supervised Data Collection

Forty PwMS (inclusion: no major health conditions affecting balance other than MS, no acute exacerbations within the previous 3-months, ambulatory without assistive devices) were recruited from the University of Vermont Medical Center Multiple Sclerosis Center and dichotomized by fall status (fallers and non-fallers). Fallers were identified with a questionnaire asking if they had sustained a fall in the previous 6-months where a fall was defined “as an event where you unintentionally came to rest on the ground or a lower level”.

Participants were instrumented with two inertial sensors (Biostamp, MC10, Inc., Lexington, MA) adhered directly to the skin; one sensor was located on the midpoint of the right thigh approximately aligned with the femur and the second sensor was located on the chest located approximately one inch distal to the sternal notch. The sensors recorded triaxial accelerometer data (sampling rate 250 Hz, ±16G) while participants performed one trial of the 30CST in which they completed sit-to-stand transitions from a 17-inch chair as quickly and safely as they felt comfortable with arms crossed over their chest. Participants completed standardized outcome measures related to fall risk [31], [32] including the Modified Fatigue Impact Scale (MFIS) [33] and the Activities-Specific Balance Confidence scale (ABC) [34]. Participant disability was quantified with the expanded disability status scale (EDSS) [35] administered by a neurologist. The EDSS pyramidal and sensory sub scores were used to dichotomize the group into additional classification schemas to investigate pyramidal and sensory impairment [36], [37]. Participants were divided into those without pyramidal impairment (rated at 0 or +1) and those with manifest clinical disability (rated above +1). Similarly, participants that were rated at 0 normal or +1 in the sensory sub score were classified as having no sensory impairment and any participants that received a score above +1 were classified as having sensory involvement with manifest clinical disability. The experimental protocol was approved by the University of Vermont IRB and all participants provided informed consent.

### B. Unsupervised Data Collection

Participants completed approximately two days of daily life unsupervised monitoring immediately following the supervised session. Prior to leaving the laboratory new sensors (sampling rate 62.5 Hz) were placed on the right thigh and chest in the same location and a 30-second static standing calibration trial was performed. The participants were provided with a smartphone and oriented to an application that interfaced with the MC10 sensors (i.e., MC10 Link App) to facilitate unsupervised data collection. The sensors are waterproof thus participants wore the sensors continuously during the unsupervised period and all data were stored in the sensors’ local memory. Accelerometer data were uploaded to a secure MC10 cloud-based database once the sensors were returned via mail, and the data were downloaded to a local server for processing. During unsupervised monitoring participants were asked to complete bi-hourly 30CSTs (timeframe 07:00 to 21:00) and an alarm was enabled on the smartphone at the requested task completion times to facilitate adherence. Study personnel provided a handout with instructions and reviewed the 30CST technique which included employing the same technique as performed during the supervised session, the type of chair to use and how the chair should be positioned against a stable surface for safety.

### C. Data Processing for 30CST Data

**Fig. 1.**
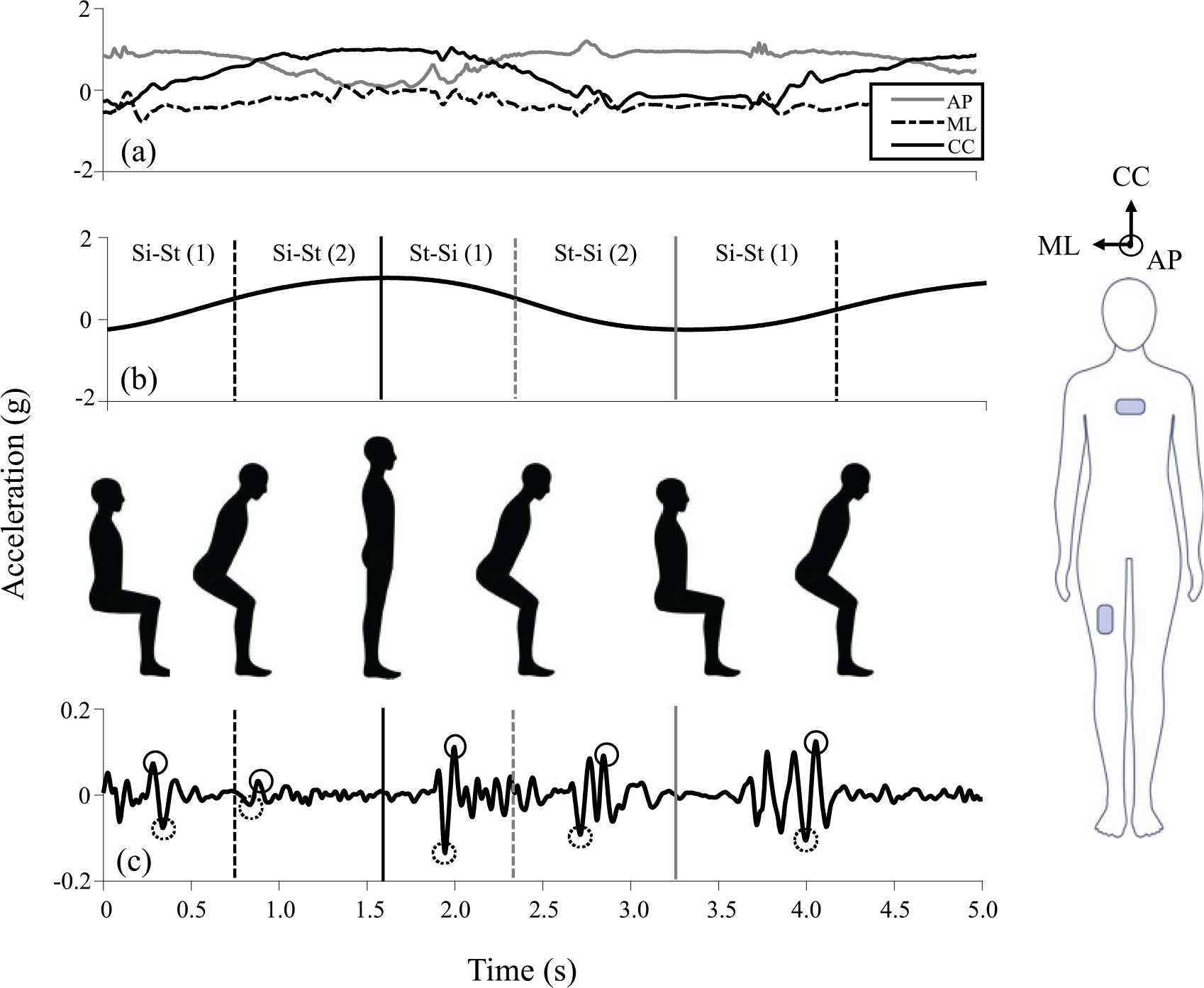
Overview of algorithm for delineating phases of the 30CST and extracting performance metrics. Triaxial accelerometer data were approximately aligned with the anterior-posterior (AP), cranial-caudal (CC), and medial-lateral (ML) directions (a) using the participant specific static standing pose (right) and direction of gravity. The CC component was low pass filtered and sit (minimum values, gray solid line), stand (maximum values, black solid line) and mid-transition (inflection points, dotted lines) events were identified to create four regions of interest (b). The triaxial raw accelerometer signal was bandpass filtered (c) and used to extract peak (solid circles) and minimum values (dotted circles) for all components for the chest and thigh.

The raw accelerometer data for all 30CSTs (supervised and unsupervised) were processed using a fully automated Matlab algorithm (Mathworks, Natick MA). The algorithm used only the thigh accelerometer signal to delineate the sit-to-stand (si-st) and stand-to-sit (st-si) phases of each 30CST repetition and has been previously detailed [27], [38]. Fig. 1 provides an overview of the algorithm. Briefly, the raw accelerometer data were projected onto participant-specific thigh reference frames using data from the static standing trial. The new reference frame was approximately aligned with the anterior-posterior (AP), medial-lateral (ML) and cranial-caudal (CC) directions (Fig. 1, right) using the known thigh sensor location and the direction of gravity. The 30CST data were low pass filtered using a 3rd order Butterworth IIR filter with a cutoff frequency equal to the dominant frequency observed during the 30CST. Sit and stand events were then identified as the minimum and maximum values, respectively, in the cranial-caudal component (Fig. 1, bottom).

### D. Performance Metrics: Supervised 30CST

Performance metrics extracted for the supervised session included temporal and acceleration-related features from each of the triaxial acceleration components. Sit and stand events extracted from the CC component of the thigh acceleration were used to compute si-st and st-si duration times for each transition. Then temporal features were computed which included the average, median, maximum, and minimum si-st and st-si transition times across all repetitions of one 30CST as well as the number of 30CST repetitions performed (nine features total). Triaxial (AP, CC and ML) peak and minimum thigh and chest accelerations were extracted within the first and second half of each 30CST transition (Fig. 1) from raw accelerometer signals bandpass filtered using a 3rd-order Butterworth IIR filter with cutoff frequencies of 5 and 20 Hz. These cutoffs were selected to limit signal content to a physiologically relevant range while also removing the effects of changes in sensor orientation. The average and median of the peak and minimum accelerations as well as the 95th percentile and 5th percentile acceleration values across all repetitions of each 30CST in the first and second halves of the sit-stand and stand-sit transitions were computed and used as performance metrics for analysis (144 features total, 72 features each for the thigh and chest). Details of this approach are provided in [38].

### E. Performance Metrics: Unsupervised 30CST

Participants had to complete a minimum of four 30CST assessments during the unsupervised period for inclusion in the analysis for statistical analysis. The raw accelerometer data for each 30CST was processed using the same algorithm as described for the supervised 30CST to delineate each 30CST assessment into si-st and st-si components. The minimum, maximum and average si-st and st-si times were computed for each 30CST. As there were varying numbers of 30CST assessments completed by each participant, the performance metrics reflected summary statistics across all the 30CST assessments performed (163 features total) and included: overall average and median of si-st time across all 30CSTs, overall average and median of st-si time across all 30CSTs, average 30CST performance (number of repetitions), average of the maximum and minimum si-st and st-si times, median of the maximum si-st and st-si times, 5th percentile si-st and st-si times across all 30CSTs, 95th percentile si-st and st-si times across all 30CSTs, and median of the average si-st and st-si times (total of 19 metrics). Similarly, the acceleration-related metrics reflected summary statistics across all 30CSTs for each component (AP, CC, ML) for the thigh and chest for the first and second halves of each si-st and st-sit transition (144 features total). For example, for the AP component of the thigh this included the average and median of the peak acceleration, average and median of the minimum acceleration and the 5th and 95th percentile acceleration value across all 30CSTs.

### F. Data Processing for Daily Life Data

**Fig. 2.**
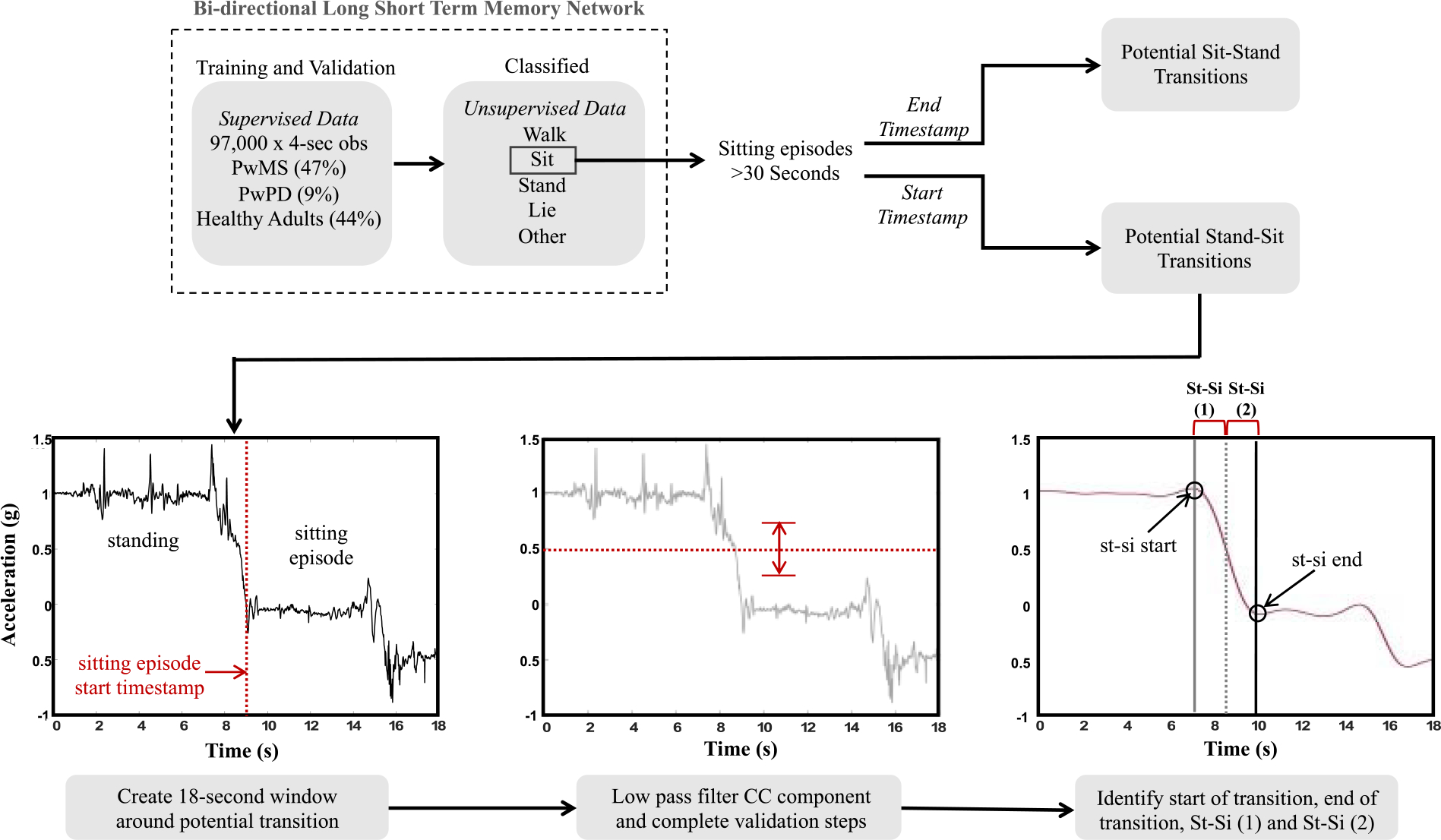
Overview of daily life sit-stand (si-st) and stand-sit (st-si) transition data processing. The example shown illustrates the pipeline for stand-sit transition but the methodology was the same for sit-stand transitions.

Fig. 2 provides an overview of the data processing steps to identify sit-stand and stand-sit transitions during daily life. Activity bouts were identified from chest and thigh wearable sensor data using a deep learning approach that leverages a Long Short Term Memory (LSTM) architecture adapted from [39]. This model was trained to identify walking, standing, sitting, lying, and “other” (i.e., non-classified activities). Specifically, the network was composed of a LSTM layer with 215 hidden units and a 30% drop out layer, followed by a BiLSTM Layer with 125 hidden units, a 40% drop out layer and Adam optimization [40]. This classifier was developed using data from 88 participants that included 47% data from PwMS (i.e., participants from this study), 44% from healthy adults, and 9% from persons with Parkinson’s Disease to provide a wide variety of example activities representative of multiple patient populations. Data labeled as walking, standing, sitting, or lying came from prescribed activities from supervised (laboratory) data collection sessions. Data labeled as “other” consisted of running, stair ascent, stair descent and unidentifiable periods of activity and approximately 1,000 entries were manually labeled. Ten-fold cross validation was conducted on the training set consisting of 97,000 4-second observations (1:1:1:1:0.85 walk:stand:sit:lie:other) yielding validation AUC of 0.99 for sitting. Data from a given participant were not included in both training and held-out test sets. Performance on a held-out test set consisting of 31,863 observations (1:1:1:1:0.66 walk:stand:sit:lie:other) achieved 96.3% accuracy overall, providing evidence that the classifier was appropriate for use on new datasets.

The model was then leveraged to identify all sitting bouts completed by participants during the 48-hour free-living period (see Fig. 2). All sitting bouts greater than 30-seconds identified by the classifier were extracted for further analysis as potential sit-stand or stand-sit transitions. 9-seconds of raw data were extracted from either side of the relevant timestamp to create windows 18-seconds in length that encompassed either the beginning or ending timestamps of each sitting bout to examine each window for a potential stand-sit or sit-stand transition, respectively. The raw accelerometer data were then processed using a fully automated Matlab algorithm (Mathworks, Natick MA). The CC-component of the raw acceleration was low pass filtered (LPF) using a 3rd order Butterworth IIR filter with a cutoff frequency equal to 0.4, which was the low range of the dominant frequency observed during the 30CST in our earlier work [38] and isolates accelerometer signal content related to the changes in body segment orientation characteristic of the STS task. The sit and stand events were identified as the minimum and peak values, respectively, closest temporally to the transition point. The potential window of signal content was passed through a series of validation steps which included:

1. the CC-component LPF signal had to pass through 0.5 g (Fig. 2),
2. the CC-component LPF signal had to demonstrate >0.5 g of total range (Fig. 2) and,
3. STS duration had to be less than 4.5 seconds [40], [41].

Any potential window not meeting these criteria were discarded from further analysis. Approximately one-third of the participants’ data were visually inspected to ensure the automated algorithm was correctly discarding windows that did not contain a transition.

### G. Performance Metrics: Daily Life Transitions

The minimum, maximum, average, median and coefficient of variation (CV) si-st and st-si times were computed across all sit-stand and stand-sit transitions identified during daily life (ten features total). Triaxial (AP, CC and ML) peak and minimum thigh and chest accelerations were extracted within the first and second half of each sit-stand and stand-sit transition similar to the 30CST data. The average, median and CV of the peak and minimum accelerations as well as the 95th percentile and 5th percentile acceleration values across all sit-stand and stand-sit transitions in the first and second halves of the sit-stand and stand-sit transitions were computed and used as the performance metrics for the analysis (128 features total).

### H. Statistical Analysis

Independent sample Student’s t-tests and Wilcoxon Rank Sum Tests (for non-normal variables determined via Kolmogorov-Smirnov test) were used to evaluate differences between fallers and non-fallers, non-pyramidal and pyramidal impairment and sensory and non-sensory impairment for all clinical outcome measures and accelerometer-derived thigh and chest performance metrics with a threshold of p<0.05. Then for all accelerometer-derived metrics demonstrating statistically significant differences between groups, non-parametric receiver operating characteristics (ROC) analyses with age-adjusted covariates were used to classify fall risk status (high/low), no pyramidal vs pyramidal impairment (NPI/PI) and no sensory vs sensory impairment (NSI/SI). The area under the receiver operator characteristic (ROC) curve (AUC) was used to quantify each performance metrics’ ability to distinguish between groups and enables a quantitative comparison across all three conditions (supervised 30CST, unsupervised 30CST, daily life) [28]. We ranked all features from highest to lowest AUC and reported those that achieved AUC > 0.70 for discriminating high and low fall risk, pyramidal impairment, and sensory impairment. Effect size was also evaluated, where appropriate, using the Cohen’s d statistic (d). Statistical analyses were performed in Stata (StataCorp LLC, College Station, TX).

## III. Results

### A. Group Characteristics

Table I summarizes the demographics for the participants. Data for three participants were disregarded due to sensor technical difficulties or non-compliance of unsupervised protocols; of the remaining 37 participants there were 21 fallers, 14 participants with pyramidal impairment and 24 with sensory impairment. Across all groups there were no differences in total hours of unsupervised monitoring, total number of 30CST assessments performed during the unsupervised monitoring period, or total number of daily life sit-stand transitions identified during the two days of unsupervised monitoring. Fallers were older and more impaired in their EDSS and ABC scores compared to non-fallers. Participants with pyramidal impairment were older, more impaired in EDSS and ABC scores and reported higher levels of fatigue. Only EDSS and MFIS scores were different for those with sensory impairment. Adherence to the unsupervised monitoring was similar between all group pairings in terms of number of hours of monitoring (cohort = 44.8 ± 5.2 hours), number of 30CST assessments performed during the unsupervised monitoring period (cohort = 12.2 ± 3.0 CSTs), and number of daily life transitions detected during the unsupervised monitoring period (cohort = 59.2 ± 17.3 transitions).

### B. Discriminating Fall Status

**Fig. 3.**
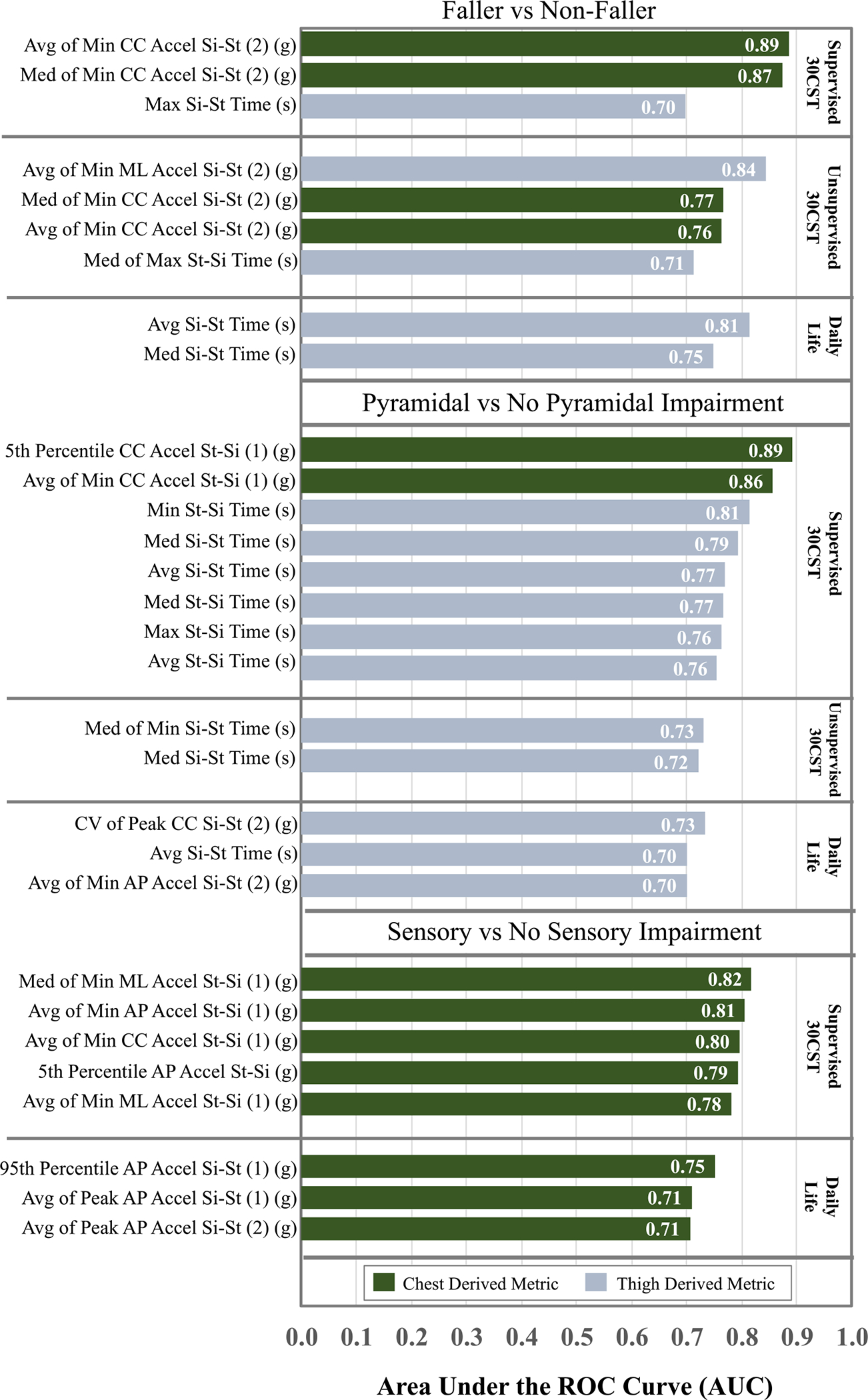
Comparison of performance metrics with AUC ≥ 0.70 for supervised 30CST, unsupervised 30CST and daily life transitions for three groups of classifiers: fallers vs non-fallers, pyramidal vs no pyramidal impairment and sensory vs no sensory impairment.

A total of 9 features (supervised 30CST = 3, unsupervised 30CST = 4, daily life = 2) including chest and thigh derived performance features (chest = 4, thigh = 5) were statistically significantly different (p < 0.05, Table II) and discriminated fall status with AUC > 0.70 (Fig. 3). Supervised 30CST performance (# reps) achieved statistical significance (p=0.026) between the groups but not AUC > 0.70 (Table II). The feature that best discriminated fallers and non-fallers was a chest feature from the supervised 30CST, the average across all repetitions of the minimum CC acceleration during the 2^nd^ half of the sit-stand transition (Avg of Min CC Accel Si-St (2), AUC = 0.89, p = 0.003, d = 0.96, Fig. 3, Table II). The daily life feature that best discriminated fall status was the average si-st time across all sit-stand transitions performed during the unsupervised monitoring Avg Si-St Time, AUC = 0.81, p = 0.0002, d = 1.15, Fig. 3, Table II).

### C. Discriminating Pyramidal Impairment

A total of 13 features (supervised 30CST = 8, unsupervised 30CST = 2, daily life = 3) discriminated between participants with and without pyramidal impairment with AUC > 0.70 (Fig. 3) and p<0.05 (Table II). The feature that best discriminated pyramidal impairment was a chest feature from the supervised 30CST, the 5th percentile of the caudal-cranial (CC) acceleration during the first half of the st-si phase (5th Percentile CC Accel St-Si (1), AUC=0.89, p=0.001, d=1.03), Fig. 3, Table II).

### D. Discriminating Sensory Impairment

Only chest derived performance features discriminated between participants with and without sensory impairment (Fig. 3, Table II). The feature that best discriminated between groups was also measured during the supervised 30CST, the median of minimum ML acceleration across all 30CST repetitions during the first half of the st-si phase (Med of Min ML Accel St-Si (1), AUC=0.82, p=0.002, d=1.07, Fig. 3, Table II).

## IV. Discussion

In this study we directly compared the ability of sit-stand and stand-sit performance metrics to discriminate impairment and fall risk in PwMS during instrumented structured and unstructured tasks in supervised settings and daily life. Our findings are consistent with recent findings in gait analysis that group discrimination is optimized by different features and is context dependent [28]. However, our work also underscores the value of supervised monitoring with structured tasks to elicit motor behavior that appears to relate to specific impairments. Additionally, acceleration features derived from the chest sensor outperformed thigh-derived (temporal and acceleration- related features to discriminate between groups in all three comparisons. (Table II, Fig. 3). While contextual differences were observed, the feature type (temporal vs acceleration-related), sensor location (thigh vs chest) and task (sit-stand versus stand-sit) all appeared to influence the ability to discriminate between groups (Table II, Fig. 3).

The instrumented 30CST as a functional assessment to identify specific modes of impairment could lead to improved ability to target and individualize therapeutic interventions prior to a fall occurring. The original (non-instrumented) 30CST has been historically associated with lower extremity functional strength in older adults [41]–[43], thus the results of our study are consistent with the 30CST as an indicator of pyramidal impairment. We expected thigh acceleration to reflect pyramidal impairment, and instead chest acceleration features during the stand-sit phase were the most robust features for discriminating pyramidal impairment. Less surprising was the role of the trunk in detecting sensory impairment. In particular, differences in the ML and AP components of trunk acceleration appear to represent deficits in postural control and balance when eccentric control and coordination are required to simultaneously direct the center of mass caudally and posteriorly to a specific target while transitioning from standing to sitting [19], [22]. Interestingly, a previous study of gait analysis investigating the use of spatiotemporal gait parameters to inform sensory/pyramidal impairment were successful in relating gait dysfunction to pyramidal but not sensory involvement [36]. Thus, task dependence may play a role in the identification of specific impairments and our inclusion of a chest acceleration metric allowed for identifying a potential movement pattern unique to sensory deficits for all three components (ML, AP, CC) and all with strong effect sizes (d>.95, Table II). Sensory inputs and biomechanical constraints, which include strength deficits, are both instrumental to a healthy balance system [10], thus instrumented functional assessments could serve to improve clinicians’ accuracy to target and then intervene on these systems.

Given the constraints of relatively short and infrequent clinical visits to observe and quantify behavior that translate to increased fall risk, we expected that performance metrics during daily life would outperform supervised performance metrics. We hypothesized that daily life metrics would capture the inherent variability associated with task performance, for example divided attention, fluctuating symptoms, and environmental factors. While sit-stand time discriminated fall risk with a relatively high AUC (AUC = 0.81, Fig. 3), only one CV metric associated with CC acceleration of the thigh (CV of Peak CC Si-St (2), Table II, Fig. 3) performed with AUC > 0.70. It is worth noting, while daily life features do not achieve the highest AUC in classification between any of the groups, the average sit to stand time in daily life showed the highest overall Cohen’s D when comparing fallers to non-fallers (see Fig. 3). This suggests that we can still find a strong difference between fallers and non-fallers in free-living conditions without prescribed tasks. Thus, a relatively short unsupervised monitoring period with a single thigh sensor after a clinic visit would be beneficial for detecting fall risk, but the timeframe presented here may not be sufficient to characterize task variability. Conversely, standardized functional tests likely reduce variability affiliated with fall risk in daily life, but still may prove useful for detecting underlying mechanisms for disability. Thus, the 30CST, and particularly an instrumented version, could serve to direct clinicians toward targeted interventions to improve strength or sensory deficits.

There were several limitations of this study. Our total sample size of 37 participants led to modest group sizes for each cohort. Additionally, two days of remote monitoring may not be enough to capture variability in this task. While we identified features with strong effect sizes, we were not able to achieve any AUC values greater than 0.90 for discriminating groups. Therefore, future work should include a larger sample from a more diverse cohort to understand if discriminative ability can be increased. A longitudinal study including interventions to target pyramidal and sensory impairment would further strengthen the utility of the 30CST to identify and assess performance metrics to measure change over time.

## V. Conclusion

There are benefits for both supervised (structured) assessments and unsupervised (daily life) monitoring of sit-stand and stand-sit transitions. The findings of this study suggest that to optimally identify fallers, pyramidal impairment, and sensory impairment, it is best to use an instrumented 30CST performed in-clinic based on this cohort of PwMS and feature set. Unsupervised sit-stand and stand-sit transitions were still able to seperate groups with high performance in classification and group difference implying that this approach could still be useful for quantifying patient symptoms outside of the clinic. Additionally, variability measures of daily life performance did not strongly distinguish between groups. Thus, when supervised monitoring is not available, a shorter unsupervised monitoring period may suffice.

## Figures and Tables

**TABLE I:** Participant Demographics and Adherence During Unsupervised

Group	Cohort *(N = 37)*	NF *(N = 16)*	F *(N = 21)*	*P*	NPI *(N=23)*	PI *(N = 14)*	*P*	NSI *(N = 13)*	SI *(N=24)*	*P*

*Demographics*										

Age (yis)	50.6±12.3	44.7±12.4	55.2±10.3	0.008	46.6±12.4	57.3±8.9	0.008	45.8±12.9	53.3±11.3	0.07
EDSS	2.6±1.4	2.0±1.0	3.1±1.5	0.01	2.0±1.0	3.6±1.4	0.0004	1.9±1.5	3.0±1.1	0.01
ABC	81.3±17.7	88.9±12.5	75.5±19.1	0.02	89.9±11.4	67.1±17.2	<0.0001	87.6±17.4	77.9±17.2	0.11
MFIS	32.8±18.8	26.9±18.8	37.3±18.0	0.10	27.3±17.5	41.9±18.0	0.02	24.1±18.6	37.5±17.6	0.04

*Unsupervised Monitoring*										

30CST Assessments (#)	12.2±3.0	11.9±3.5	12.5±2.6	0.58	11.8±3.4	12.9±1.6	0.28	13.5±1.2	11.7±3.4	0.11
Hours Monitoring (hrs)	44.8±5.2	45.4±3.0	44.3±6.4	0.54	43.9±6.5	46.1±0.3	0.22	46.2±0.3	44.0±6.4	0.23
Daily Life Transitions (#)	59.2±17.3	59.6±20.4	58.9±15.0	0.90	59.5±20.6	58.5±10.4	0.87	61.5±16.9	57.9±17.7	0.55

NF: non-faller, F: faller, NPI: no pyramidal impairment, PI: pyramidal impairment, NSI: no sensory impairment, SI: sensory impairment, EDSS: Expanded Disability Status Scale, MFIS: Modified Fatigue Impact Scale, ABC: Activity Balance Confidence, 30CST: 30-second chair stand test. P-values were based on independent sample Student’s T-tests comparing groups.

**TABLE II:** Comparison of Performance Metrics Derived from Supervised 30CSTS, Unsupervised 30CSTS and Daily Life Grouped by Classifier

	Performance Metric	*Avg ± SD*	*Avg ± SD*	*p*	*d*

	*Falls: F vs NF*	NF *(N=16)*	F *(N=21)*		

Supervised 30CST	Avg of (Chest) Min CC Accel Si-St (2) (g)	−0.06 ± 0.04	−0.03 ± 0.01	0.003	0.96
	Median of (Chest) Min CC Accel Si-St (2) (g)	−0.05 ± 0.03	−0.03 ± 0.01	0.002	0.99
	Max Si-St Time (s)	1.36 ± 0.27	1.74 ± 0.52	0.011	0.84
	*30CST Performance (# Reps)	13.1 ± 3.13	10.9 ± 2.35	0.026	0.75

Unsupervised 30CST	Avg of (Thigh) Min ML Accel Si-St (2) (g)	−0.13 ± 0.06	−0.085 ± 0.03	0.006	0.91
	Median of (Chest) Min CC Accel Si-St (2) (g)	−0.06 ± 0.02	−0.04 ± 0.02	0.008	0.88
	Avg of (Chest) Min CC Accel Si-St (2) (g)	−0.06 ± 0.02	−0.04 ± 0.02	0.014	0.83
	Median of Max St-Si Time (s)	1.16 ± 0.22	1.44 ± 0.40	0.021	0.77

Daily Life	Avg Si-St Time (s)	2.86 ± 0.10	3.04 ± 0.15	0.0002	1.15
	Med Si-St Time (s)	2.80 ± 0.12	2.94 ± 0.15	0.004	0.91

	*Pyramidal: NPI vs PI*	*NPI (N=23)*	*PI (N=14)*		

Supervised 30CST	5th Percentile (Chest) CC Accel St-Si (1) (g)	−0.14 ± 0.07	−0.067 ± 0.03	0.001	1.03
	Avg of (Chest) Min CC Accel St-Si (1) (g)	−0.08 ± 0.04	−0.05 ± 0.02	0.002	0.98
	Min St-Si Time (s)	1.08 ± 0.27	1.38 ± 0.26	0.001	1.00
	Median Si-St Time (s)	1.18 ± 0.27	1.49 ± 0.32	0.002	0.95
	Avg Si-St Time (s)	1.18 ± 0.26	1.52 ± 0.33	0.001	0.99
	Median St-Si Time(s)	1.21 ± 0.28	1.50 ± 0.26	0.002	0.95
	Avg St-Si Time (s)	1.21 ± 0.28	1.51 ± 0.27	0.002	0.95
	Max St-Si Time (s)	1.35 ± 0.33	1.66 ± 0.31	0.004	0.87
	*30CST Performance (# reps)	13.0 ± 2.80	10.0 ± 1.90	<0.001	1.03

Unsupervised 30CST	Median of Min Si-St Time (s)	0.94 ± 0.20	1.23 ± 0.35	0.004	0.97
	Median Si-St Time (s)	1.05 ± 0.21	1.38 ± 0.40	0.003	1.00

Daily Life	CV of Peak (Thigh) CC Si-St (2) (g)	0.90 ± 0.23	1.12 ± 0.26	0.010	0.85
	Avg Si-St Time (s)	2.93 ± 0.14	3.03 ± 0.16	0.047	0.67
	Avg of (Thigh) Min AP Accel Si-St (2) (g)	−0.47 ± 0.20	−0.36 ± 0.12	0.050	0.64

	*Sensory: NSI vs SI*	*NSI (N=11)*	*SI (N=29)*		

Supervised 30CST	Median of (Chest) Min ML Accel St-Si (1) (g)	−0.038 ± 0.01	−0.026 ± 0.01	0.002	1.07
	Avg of (Chest) Min AP Accel St-Si (1) (g)	−0.056 ± 0.03	−0.035 ± 0.01	0.005	0.99
	Avg of (Chest) Min CC Accel St-Si (1) (g)	−0.085 ± 0.04	−0.054 ± 0.02	0.007	0.95
	5th Percentile (Chest) AP Accel St-Si (1) (g)	−0.092 ± 0.05	−0.056 ± 0.02	0.006	0.96
	Avg of (Chest) Min ML Accel St-Si (1) (g)	−0.041 ± 0.02	−0.029 ± 0.01	0.005	0.98
	*30CST Performance (# Reps)	13.5 ± 3.39	11.1 ± 2.36	0.020	0.83

Daily Life	95th Percentile AP Accel Si-St (1) (g)	0.383 ± 0.18	0.830 ± 0.63	0.018	0.80
	Avg of Peak AP Accel Si-St (1) (g)	0.178 ± 0.08	0.424 ± 0.37	0.023	0.77
	Avg of Peak AP Accel Si-St (2) (g)	0.201 ± 0.10	0.417 ± 0.36	0.043	0.69

* Included for reference to current standard of care (counting repetitions performed in 30 seconds).

F: fallers, NF: non-fallers, NPI: minimal pyramidal impairment, PI: pyramidal impairment, NSI: minimal sensory impairment, SI: sensory impairment CC: cranial-caudal, ML: medial-lateral, AP: anterior-posterior, 30CST: 30-second chair stand test, reps: repetitions, si-st: sit-to-stand, st-si: stand-to-sit. P-values represent group comparisons based on evaluated using Cohen’s *d*.
